# Development and evaluation of the Singapore Caregiver Quality of Life Scale - Dementia

**DOI:** 10.1186/s41687-020-00252-3

**Published:** 2020-10-19

**Authors:** Yin Bun Cheung, Irene Teo, Wee Shiong Lim, Allyn Hum, Shirlyn H. S. Neo, Grace M. Yang, Geok Ling Lee, Gretchen Tan, Dennis C. C. Seow

**Affiliations:** 1 Program in Health Services & Systems Research and Centre for Quantitative Medicine, Duke-NUS Medical School, Level 6, Academia, 20 College Road, Singapore, 169856 Singapore; 2 Centre for Child Health Research, Tampere University, Tampere, Finland; 3 Lien Centre for Palliative Care, Duke-NUS Medical School, Singapore, Singapore; 4 Division of Supportive and Palliative Care, National Cancer Center, Singapore, Singapore; 5 Department of Geriatric Medicine, Institute of Geriatrics and Active Aging, Tan Tock Seng Hospital, Singapore, Singapore; 6 Department of Palliative Medicine, Tan Tock Seng Hospital, Singapore, Singapore; 7 Department of Social Work, Faculty of Arts and Social Sciences, National University of Singapore, Singapore, Singapore; 8 Department of Geriatric Medicine, Singapore General Hospital, Singapore, Singapore

**Keywords:** Caregivers, Dementia, Measurement scale, Quality of life

## Abstract

**Purpose:**

To develop and evaluate a measurement scale for multi-domain assessment of the quality of life of family caregivers of persons with dementia (PWD) in Singapore, a multi-ethic society in South-East Asia where English is the lingua franca.

**Methods:**

Items from the Singapore Caregiver Quality of Life Scale (SCQOLS), which was originally developed in the context of advanced cancers, were adopted as candidate items. Furthermore, a multi-disciplinary panel reviewed dementia-specific caregiver quality of life scales to identified items not covered in SCQOLS for inclusion as candidate items. A pilot study of 31 family caregivers of PWD was conducted to solicit inputs on candidate items; 102 family caregivers of PWD were surveyed for evaluation of the scale’s measurement properties.

**Results:**

Factor analysis confirmed a 5-domain structure of the 63 candidate items. The Root Mean Square Error of Approximation was 0.056 and Comparative Fit Index was 0.928. Convergent validity of the total and domain scores was demonstrated in terms of correlation with the Brief Assessment Scale for Caregivers and its sub-scales. The scores also showed an expected pattern of correlation with hours spent on caregiving per week. Known-group validity was demonstrated by differences in mean scores between functional staging groups. Cronbach’s alpha of the total and domain scores ranged from 0.89 to 0.95. Test-retest reliability (intraclass correlation coefficient) ranged from 0.77 to 0.92.

**Conclusions:**

The Singapore Caregiver Quality of Life Scale – Dementia (SCQOLS-D) is a quality of life measurement scale for family caregivers of persons with dementia that is valid and reliable.

## Introduction

Dementia is a chronic debilitating disease that impacts on not only physical and cognitive functions of persons with dementia (PWD) but also quality of life (QOL) of their family caregivers (caregivers in short). As the global population ages, the incidence of dementia is on the increase. It is estimated that there will be over 39 and 70 million PWD in the Asia-Pacific region by 2030 and 2050, respectively [1]. Furthermore, changing socio-demographic patterns such as smaller family size will pose further challenges to caregiving by family members.

While dementia is not entirely different from other chronic diseases in terms of impact on caregivers, it does have unique features. As examples, caregivers of PWD may suffer the feeling of their loved ones becoming strangers to them or embarrassment related to their loved one’s behavior that arises from the condition [2, 3]. Caregivers of PWD, compared to caregivers of patients with other illnesses, are at risk of higher caregiver burden [4]. Effective and ethical management of dementia should involve both the patient and caregiver [5, 6]. In the Asian culture, it is expected that people who are ill should be cared for by their family members as much as possible [7]. In the USA, Asian American caregivers provided more caregiving hours than caregivers of other ethnicity [8].

Generic QOL measurement scales that were developed for patients or the general public, such as WHOQOL-BREF, EQ-5D and SF-36, have been used to assess caregivers of PWD. However, they consider neither the special features of dementia nor the impact of chronic diseases on caregivers. From the literature we identified three dementia-specific caregiver QOL scales. The Alzheimer’s Carer’s Quality of Life Inventory (ACQLI) [9] and the caregiver QOL scale in the PIXEL dementia study [10] were both developed in Europe. Similar to caregiver burden measures, they focus on the negative experience of caregiving. However, previous studies have shown that “caregiver burden and caregiver well-being are not opposite sides of the same coin” [11, 12]. As such, these measures do not sufficiently capture caregiver QOL. The Caregiver-targeted Quality of Life Measure (CGQOL) for caregivers of PWD was developed in the USA [13]. While this 80-item scale has fairly comprehensive content coverage, it does not have a physical well-being domain and its concept of spirituality differs from the primarily existential aspects of spirituality found in Singapore, a multi-ethnic society in South-East Asia where English is the lingua franca [14].

Few QOL measurement scales were originally developed in Asia. Differences in socio-cultural context can affect the measurement of QOL in dementia [15]. Our previous study of family caregivers of patients with advanced cancer in Singapore has shown substantial differences between the concerns of caregivers here and the contents of caregiver QOL measurement scales that were developed in the West [14]. For example, positive mental health aspects such as “feeling appreciated” are missing from the QOL measurement scales, but they were found to feature prominently among Singaporean caregivers. This appears to reflect the concept of “relational self” in Asian culture [16]. Furthermore, the existential aspects of spirituality stood out in the Singaporean caregivers [14]. These findings contrast with the more individualistic focus on positive emotions and religious focus of spirituality measured by some QOL measurement scales developed in the West. Similar concerns have been reported in other Asian countries. For example, a study in China evaluated the Chinese version of the Caregiver Quality of Life Index – Cancer. It found “only partial support for the relevance and construct validity of the scale for Chinese caregivers” [17]. Therefore, we recently developed and validated a locally derived and culturally appropriate QOL measurement scale for caregivers of patients with advanced cancers: the Singapore Caregiver Quality of Life Scale (SCQOLS) [18]. The items were generated with caregiver inputs through qualitative research [14]; the scale was then evaluated in a quantitative study and found to be valid and reliable [18].

Although the SCQOLS was originally developed in the context of advanced cancer, in our literature review we have found that most of the items in the dementia-specific caregiver QOL scales (CGQOL, ACQLI and PIXEL) are represented by the SCQOLS, reflecting the commonality of the impact of many chronic diseases. However, some of their items are not covered by SCQOLS, such as feeling that the family member with dementia has become a stranger and feeling embarrassed by their behavior. These issues are specific and important for the caregivers of PWD and should not be ignored. Therefore, we conducted this study to develop and validate a QOL measurement scale in the English language for caregivers of PWD.

## Methods

### Study setting

Singapore is a multi-ethnic society, with Chinese (74%), Malays (13%) and Indians (9%) being the major ethnic groups according to the 2010 census [19]. A pilot study was conducted in the Department of Geriatric Medicine of Singapore General Hospital and Department of Palliative Medicine of Tan Tock Seng Hospital, followed by a validation study conducted in the Department of Geriatric Medicine of Singapore General Hospital. Both studies were approved by the Singapore Health Services Centralized Institutional Review Board (#2017/2607 and #2018/2896). Informed consent was obtained from all individual participants included in the study.

### Questionnaire development

The Singapore Caregiver Quality of Life Scale (SCQOLS) comprises five domains, namely Physical Well-being (PW; 12 items), Mental Well-being (MW; 10 items), Experience & Meaning (EM; 12 items), Impact on Daily Life (DL; 13 items) and Financial Well-being (FW; 4 items). The items were rated on a 5-point scale. They were included as candidate items.

A panel consisting of six investigators, including a geriatrician (WSL), a clinical psychologist (IT), a social worker (GLL), two palliative medicine physicians (SHN and GMY) and a health outcomes researcher (YBC) reviewed three caregiver QOL measures in dementia, namely, ACQLI, PIXEL and CGQOL [9, 10, 13], to map their items to the corresponding items in SCQOLS and identify remaining items not captured by SCQOLS for inclusion as candidate items. The mapping exercise began with each investigator individually reviewing each item in ACQLI, PIXEL and CGQOL. For each item, they made note on whether they found item(s) in the SCQOLS that covered the same or similar concept, whether they would recommend the inclusion of the item into the draft questionnaire, and the reason of their recommendation. After that was completed, the panel jointly reviewed the results. For items that did not reach consensus in the individual recommendations, the panel discussed the rationale until a consensus to include or not was reached. No formal decision making guideline was established a priori.

### Pilot study

For both the pilot and validation studies, we defined a family caregiver as a family member who was taking direct care of the patient’s day-to-day and healthcare needs, or ensuring provision of care to meet the needs, or who was the decision maker with regard to the patient’s needs and healthcare. Participants must be living with the PWD or spent at least 10 h per week in giving care to the PWD and aged at least 21 years.

We recruited 31 English-speaking caregivers, whose PWD care recipients were receiving care from the study centers. The draft version of the caregiver QOL scale was administered. Open-ended questions were included in the questionnaire package to seek feedback on the readability and relevancy of the questions and on whether there were other important QOL concerns that should be added to the scale.

The panel members jointly reviewed the feedback in the pilot study and discussed and shared their rationale on why the feedback should lead to a revision/addition to the questionnaire, until a consensus to incorporate or not was reached. No formal a priori decision rules or thematic analysis was employed.

### Validation study

#### Study design and measurements

The study comprised a baseline and a follow-up survey of caregivers of PWD. Participant eligibility criteria were the same as those in the pilot study. The baseline survey included the pilot-tested caregiver QOL scale, the Brief Assessment Scale for Caregivers (BASC), which includes a Negative Personal Impact (NPI) and a Positive Personal Impact (PPI) sub-scale [20], two items on financial concerns from a modified version of the Caregiver Reaction Assessment (CRA) for use in Singapore [21], and a section on caregiver’s demographic and caregiving characteristics. The sum of the CRA item scores was referred to as CRA (Finance) in this report for brevity. The section on caregiver characteristics included a question on the number of hours spent on caregiving per week and a question that asked the caregivers whether s/he was “the only person”, “the primary person” or “one of the few persons” who carries out caregiving duties for the patient. The responses were coded as 1, 2 and 3, respectively, and the variable was treated as ordinal. For brevity, we refer to this variable as “caregiver role”. In addition, the Functional Assessment Staging Test (FAST) was administered by a research assistant [22]. These variables were used as criteria for the evaluation of the scale’s validity. Consented caregivers were invited to self-administer the questionnaire. Four caregivers requested interviewer-administration. They were not included in the present analysis.

The follow-up survey comprised the caregiver QOL scale and a question on the caregiver’s self-perceived change in QOL since the baseline survey on a 7-point scale [23]. The questionnaire together with a postage-paid return envelope was sent to the caregivers about 1 week after the baseline interview. A reminder was sent if the questionnaire was not returned within 2 weeks from the posting.

#### Statistical analysis

All the QOL items and the items in the BASC and CRA (Finance) were recoded so that a higher score indicated a better outcome.

The SCQOLS was previously established to comprise five domains. Based on the multi-disciplinary panel’s evaluation on the face validity, the additional items identified from the literature on dementia and pilot study either belong to the MW (8 items) or EM (4 items) domains. We conducted confirmatory factor analysis (CFA) of this 5-factor model, with each item loaded on one factor. The CFA was implemented using the Weighted Least Squares method for data with Missing Values (WLSMV) in Mplus [24]. The Root Mean Square Error of Approximation (RMSEA) and Comparative Fit Index (CFI) were used for assessment of goodness-of-fit [25, 26].

Upon finding a satisfactory factor structure, the simple mean imputation was used to replace item non-responses [27]. The QOL domain scores were calculated as the mean of the scores within the domains, which were on the 0 to 4 scale, and then multiplied by 25 to re-scale them to the 0 to 100 scale. The QOL total score was a weighted mean of the QOL domain scores, using the number of items in the domains as the weights.

Spearman’s correlation coefficient was calculated between the QOL scores and the validity criterion variables. Furthermore, a recent cohort study of caregivers in Singapore has demonstrated that there was no correlation between mental well-being and caregiving hours in the cohort as a whole, but there was a negative correlation among cohort members who had low level of self-competency and sense-making [28]. Given the similarity of the Experience & Meaning (EM) domain and self-competency and sense-making, we also estimated the Spearman’s correlation coefficient between QOL total and domain scores (except EM) and caregiving hours among caregivers who had EM scores below the mean. We also estimated Spearman’s correlation coefficients between each item and its belonging domain and other domains, to check if the former correlation was stronger than the latter.

Known-group validity were assessed by differences in mean QOL scores between caregivers of patients with mild (FAST 5 or below) and more severe (FAST 6a or above) functional limitation, with two-sample t-tests. Cronbach’s alpha (α) was used to determine internal consistency.

Participants who completed the follow-up survey within 21 days of the baseline survey and who had reported no change in self-perceived QOL were included in test-retest reliability assessment, using the intraclass correlation coefficient (ICC). The inclusion criteria aimed to rule out changes in QOL scores due to sources other than reliability issues.

#### Sample size determination

A minimum sample size of 100 participants provides 80% power, at 5% type 1 error rate, to reject a null hypothesis of sufficient goodness-of-fit as quantified by RMSEA ≤0.05 in confirmatory factor analysis [29]. This sample size is also sufficient for assessment of validity, with 80% power, at 5% type 1 error rate, to test a correlation coefficient of 0.3 between QOL scores and validity criteria. With reference to a previous study [18], we expected that about half of the baseline participants would respond to the follow-up survey within the target time frame. It is sufficient because a sample size of 34 gives precision (width of 95% CI) of ±0.1 in the estimation of ICC, assuming a true value of about 0.85 (PASS 13 Software).

## Results

### Item generation and pilot study

The multi-disciplinary panel identified 7 items from the CGQOL and 1 item each from ACQLI and PIXEL that were not covered in SCQOLS (Table 1). Therefore, a draft questionnaire with a total of 60 QOL items was pilot-tested.

**Table 1:** Candidate items identified from review of dementia literature and pilot study

Source^a^	Domain^b^	Items
CGQOL	MW	I worry that someone would take advantage of my relative with dementia
CGQOL	MW	I worry that my relative with dementia would do something unsafe or harm himself or herself
CGQOL	MW	I am embarrassed by the behaviour of my relative with dementia
CGQOL	MW	I worry that I might be unable to take care of my relative with dementia in the future
ACQLI	MW	I feel like my relative with dementia has become a stranger to me
CGQOL	MW	I feel that my relative with dementia is a burden to me
PIXEL	EM	I have received useful information from healthcare professionals regarding my relative’s condition
CGQOL	EM	My faith helps me cope with the challenges of caregiving
CGQOL	EM	Taking care of my relative with dementia has brought us closer
Pilot study	MW	Societal attitude towards dementia makes my caregiving difficult
Pilot study	MW	I worry that my relative with dementia would fall and hurt himself or herself
Pilot study	EM	There is sufficient practical support in the community

^a^ *ACQLI* Alzheimer’s Carer’s Quality of Life Inventory [9], *CGQOL* Caregiver-targeted Quality of Life Measure [13], *PIXEL* Pixel Dementia Study Caregiver Quality of Life Scale [10]

^b^ Initial domain proposed for confirmatory factor analysis. *MW* Mental Well-being, *EM* Experience & Meaning

Thirty-one caregivers of PWD were recruited in the pilot study. The median age was 58; 19 (61%) were female; 24 (77%) were adult children of the PWD; 25 (81%) were ethnic Chinese. They found the draft questionnaire items relevant to their concerns. However, in relation to the PIXEL item on “received adequate information”, one caregiver commented that receiving information with medical terminology is stressful and not necessarily helpful. Therefore, we changed this item to “received useful information” (Table 1). They raised three additional themes of concerns: (1) availability of respite service and foreign domestic worker (seven caregivers), (2) stigma and societal acceptance (two caregivers), and (3) worries about fall and injuries (one caregiver). Accordingly, we generated three more items for the draft questionnaire: “there is sufficient practical support in the community”, “societal attitude towards dementia makes my caregiving difficult”, and “I worry that my relative with dementia would fall and hurt himself or herself” (Table 1). The study team developed the wording of the new items with a view to succinctly summarize the concerns of the pilot study respondents. For example, two respondents voiced their concerns using the words “stigma” and “acceptance”. The team decided to use the phrase “society attitude towards dementia makes my caregiving difficult” for summarizing the concerns instead of developing two separate items. To be consistent with the other items, a five-point scale was used for the new items. These three items were not further tested before the main survey. Therefore, the questionnaire that entered the validation study comprised 63 items.

### Validation study

#### Participants characteristics

One hundred and two participants were recruited and self-administered the baseline questionnaire package. Demographic and caregiving characteristics of the participants are shown in Table 2. Most of the participants were female (80.4%), adult children (86.3%) of the PWD, and ethnic Chinese (88.2%). Half of the PWD they cared for had mild functional limitations (FAST 5 or below).

**Table 2:** Participant characteristics (*N* = 102)

Characteristics	Mean (SD) or N (%) ^a^
Age (years)	55 (11)
Gender	
Female	82 (80.4%)
Male	20 (19.6%)
Ethnicity	
Chinese	90 (88.2%)
Malay	5 (4.9%)
Indian	6 (5.9%)
Others	1 (1.0%)
Education	
Primary or below	3 (2.9%)
Secondary	27 (26.5%)
Post-secondary	72 (70.6%)
Relationship with patient	
Spouse	5 (4.9%)
Son or daughter	88 (86.3%)
Others relatives	9 (8.8%)
Hours caregiving per week	44 (22)
Caregiver role	
Only person	21 (20.6%)
Primary person	31 (30.4%)
One of the few persons	50 (49.0%)
Functional Assessment Staging (FAST) of PWD	
Mild (1 to 5)	51 (50%)
Moderate-to-severe (6a to 7f)	51 (50%)

^a^Mean and standard deviation (SD) for continuous variables; frequency (N) and percent for categorical variables

#### Factor analysis and descriptive summary

The 5-factor model gave RMSEA 0.056 (90% confidence interval 0.049 to 0.061) and CFI 0.928. All items had factor loadings ≥0.3 (Online Supplemental Material S1).

Table 3 presents the scores on the QOL scale. The mean domain scores ranged from 57 to 74. There was little floor or ceiling effects, except that 28.4% of the participants reached the ceiling of the FW domain score. The domain scores had moderate correlation among themselves (each *P* < 0.01), with the exception of EM, which has weak correlation with the other domain scores.

**Table 3:** Descriptive summary and correlation matrix of quality of life scores

Scale ^a^	Mean (SD)	N (%) Floor	N (%) Ceiling	Correlation				
				PW	MW	EM	DL	FW
PW	74 (17)	0 (0.0)	1 (0.1)					
MW	67 (16)	0 (0.0)	1 (0.1)	0.58**				
EM	57 (18)	0 (0.0)	0 (0.0)	0.21*	0.18			
DL	71 (22)	0 (0.0)	6 (5.9)	0.56**	0.62**	0.17		
FW	70 (28)	3 (2.9)	29 (28.4)	0.48**	0.49**	0.18	0.53**	
QOL Total	67 (13)	0 (0.0)	0 (0.0)	0.74**	0.80**	0.49**	0.82**	0.68**

** *P* < 0.01; * *P* < 0.05

^a^*PW* Physical Well-being, *MW* Mental Well-being, *EM* Experience & Meaning, *DL* Impact on Daily Living, *FW* Financial Well-being, *QOL Total* QOL total score

#### Validity

Table 4 shows the Spearman’s correlation coefficients between the QOL total and domain scores and various validity criterion variables. The QOL total and domain scores correlated significantly with the BASC total score, with coefficients ranging from 0.37 to 0.79 (each *P* < 0.01). The QOL total and domain scores were correlated with Negative Personal Impact, with the exception of EM. All the QOL scores, including EM, were correlated with Positive Personal Impact (each *P* < 0.05). While other domains had limited association with CRA (Finance), the Financial Well-being domain score was strongly correlated with it (0.72; *P* < 0.01).

**Table 4:** Correlation with validity criterion measures

Measures^a^	PW	MW	EM	DL	FW	QOL Total
BASC Total	0.60**	0.64**	0.37**	0.71**	0.52**	0.79**
NPI	0.31**	0.59**	0.05	0.52**	0.35**	0.51**
PPI	0.46**	0.57**	0.23*	0.47**	0.42**	0.60**
CRA (Finance)	0.27**	0.28**	0.19	0.32**	0.72**	0.43**
Caregiving hours	−0.14	−0.16	0.26**	−0.25*	−0.12	−0.10
Caregiving hours, sub-group^b^	−0.22	−0.18	N.A.	− 0.36**	− 0.16	− 0.28*
Caregiver role	0.23*	0.09	−0.03	0.22*	0.18	0.16

** *P* < 0.01; * *P* < 0.05

^a^*PW* Physical Well-being, *MW* Mental Well-being, *EM* Experience & Meaning, *DL* Impact on Daily Living, *FW* Financial Well-being, *QOL Total* QOL total score, *BASC* Brief Assessment Scale for Caregivers, *Total* Total score, *NPI* Negative Personal Impact (Factor 1 of BASC), *PPI* Positive Personal Impact (Factor 2 of BASC), *CRA (Finance)* Sum of scores on two finance items of the modified Caregiver Reaction Assessment. Caregiver role: being the only person (1), primary person (2) or (3) one of the few persons who carry out caregiving duties. Scale scores were recoded such that a higher score means a better outcome

^b^Sub-group analysis of 56 caregivers whose EM score were below the mean

Spending more hours on caregiving per week was negatively correlated with DL (− 0.25; *P* < 0.05) and positively correlated with EM (0.26; *P* < 0.01; Table 4). PW, MW, DL, FW and QOL total scores all had stronger negative correlation with caregiving hours among caregivers who had EM scores below the mean than in the whole sample. The probability of this pattern of all five scores differing in the same direction between the whole sample and sub-sample analyses occurring by chance was *P* = 0.5^5^ = 0.031. Having family members to share caregiving duties (caregiver role) was positively correlated with PW and DL (0.23 and 0.22, respectively; each *P* < 0.05).

**Fig. 1:**
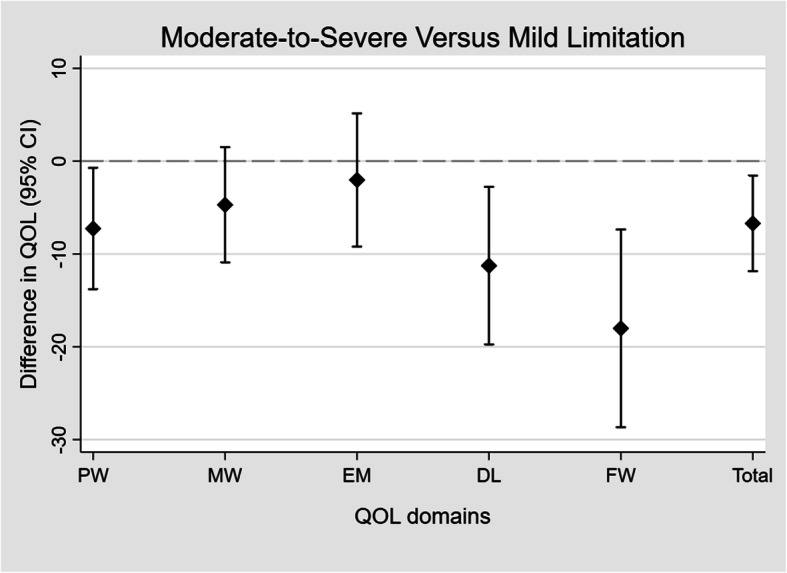
Differences in mean quality of life scores between caregivers of PWD whose care recipients had moderate-to-severe (FAST 6a or above) and mild functional limitation (FAST 5 or below). PW: Physical Well-being; MW: Mental Well-being; EM: Experience & Meaning; DL: Impact on Daily Living; FW: Financial Well-being; Total: QOL total score

Comparing the group of caregivers whose family member had mild functional limitation (FAST 5 or below) versus the group with more severe limitation (FAST 6a or above), the latter group had a mean deficit of 7, 11, 18 and 7 points in PW (*P* < 0.05), DL, FW and QOL total score (each *P* < 0.01), respectively (Fig. 1).

Correlation analysis was also conducted assess if the items were more associated with their belonging domains than the other domains. With only two exceptions, all the items exhibited a higher correlation with its belonging domain than with the other domains. The exceptions were: (1) MW14 (embarrassed) had a correlation of 0.29 and 0.31 with MW and EM, respectively (test of difference in correlation coefficient: *P* = 0.85), and (2) DL11 (disagreements with family) had a correlation of 0.44, 0.45 and 0.45 with DL, PW and MW, respectively (test of difference in correlation coefficient, DL11 with DL as reference coefficient: *P* = 0.97 [DL11 with PW] and 0.95 [DL11 with MW]. Details are provided in Online Supplemental Material S2.

#### Internal consistency and test-retest reliability

Cronbach’s alpha of the QOL scale and its 5 domains ranged from 0.89 (MW) to 0.95 (QOL total score) (Table 5).

**Table 5:** Internal consistency (Cronbach’s alpha, α) and test-retest reliability (intraclass correlation coefficient, ICC)

Scale^a^	α	ICC
PW	0.90	0.86
MW	0.89	0.89
EM	0.90	0.77
DL	0.94	0.87
FW	0.92	0.87
QOL Total	0.95	0.92

^a^*PW* Physical Well-being, *MW* Mental Well-being, *EM* Experience & Meaning, *DL* Impact on Daily Living, *FW* Financial Well-being, *QOL Total* QOL total score

A total of 49 caregivers completed the follow-up questionnaire within 21 days. Among them, 35 reported no change in self-perceive quality of life and were included in the test-retest reliability assessment. The ICC’s ranged from 0.77 (EM) to 0.92 (QOL total score) (Table 5).

## Discussion

We have developed a multi-domain caregiver QOL measurement scale for caregivers of PWD. We name this the Singapore Caregiver Quality of Life Scale – Dementia (SCQOLS-D). The identification of the item candidates involved participants’ and professionals’ inputs. The scale covers general themes that were originally developed with caring for advanced cancer patients in view, but now has been extended to cover dementia-specific concerns.

We have demonstrated that the QOL scale and its 5 domains had sufficient level of measurement properties. The RSMEA and CFI demonstrated goodness-of-fit of the 5-factor model [25, 26]. None of the items were redundant in terms of factor loading < 0.3. The validity of the domain and total scores was demonstrated by correlation with the BASC and its subscales and the CRA (Finance). The validity of PW, DL, FW and QOL total scores was also demonstrated in relation to objective measurements, i.e. hours spent in caregiving per week, caregiver role and level of functional limitation. The stronger correlation between QOL scores and caregiving hours among those with below average EM scores than in the whole sample was as predicted by the previous study that showed caregivers who reported lower level of self-competency and sense-making tended to suffer more impact [28]. We note that MW was only weakly associated with the objective measurements (each *P* > 0.05), but it correlated well with the psychometric measurements, suggesting the subjective nature of mental well-being. The EM domain covers some items that are lacking in some other caregiver QOL measures, such as feeling appreciated and feeling competent. It is different from the other domains in that it concentrates on resilience and sense making rather than problems. It had no correlation with Negative Personal Impact, but it was correlated with Positive Personal Impact. This pattern was expected due to its nature. It was positively correlated with number of hours spent taking care of PWD per week. This suggests that caregivers who had positive and meaningful experience tended to spend more time on caregiving. The internal consistency and test-retest reliability were also satisfactory for all the domain and QOL scores. The Cronbach’s alpha value was 0.95 for the total scale and between 0.89 and 0.94 for the domains. Fayers and Machin suggested that 0.9 is excellent for group level analysis and needed for making decisions about individuals [30]. The scale and domains have sufficient internal consistency for group level analysis and some of them are possibly suitable for individual decision making.

A limitation of the present study is that the follow-up survey was planned to assess test-retest reliability. Due to the short follow-up duration in the study design, we have not assessed the scale’s sensitivity to change. A second limitation of our study is that we did not collect information about foreign domestic worker. In a qualitative study of 16 caregivers whose PWD had never attended day care service, 9 (56%) employed a foreign domestic worker [31]. Employed domestic workers may help with the provision of care and therefore affect the patterns of associations in the validity analysis, especially in relation to the variable on caregiver role. The Singapore 2010 Census shows that among residents aged 15 or over, 80% are literate in English [19]. This study of English speakers and development of a new measurement scale in English covers a large part of the population. Further development and research of the scale in other languages will enhance research in and care for this population. The scale was developed for the assessment of family caregivers, not caregivers in general. The scale should not be used in other caregivers without further evaluation.

Despite the name Singapore Caregiver Quality of Life Scale – Dementia (SCQOLS-D), the scale shares some common items with caregiver QOL scales developed in other countries. Furthermore, Singapore shares similar socio-cultural background with other Asian countries, as a large proportion of the population is immigrants or their offspring: In 1921 and 2010, respectively, 71% and 43% of the population was foreign-born, mostly in Asian countries [32]. We anticipate that the scale contents are relevant to other countries, especially in Asia. We hypothesize that the scale has sufficient level of measurement performance for use in other countries. This hypothesis will need evaluation in further studies. Moreover, previous studies have demonstrated that it is feasible to empirically remove some items from an original version of a psychometric measurement scale without substantial information loss [33]. The next step for SCQOLS-D will include the generation of a short form.

In conclusion, a multi-domain quality of life measurement scale for the assessment of family caregivers of persons with dementia has been developed in Singapore. Validity and reliability of the scale have been demonstrated. It has the potential to facilitate clinical assessment, service evaluation and research in Singapore and other societies with similar socio-cultural background.

## Supplementary information

**Additional file 1: Online Supplementary Material S1.** Factor loadings in confirmatory factor analysis of a 5-factor model, with each item loaded on one factor.

**Additional file 2: Online Supplementary Material S2.** Spearman’s correlation coefficients between each item and its belonging domain and other domains.

## Data Availability

The dataset is not publicly available due to IRB restrictions but are available from the corresponding author on reasonable request.

## Abbreviations

ACQLI: Alzheimer’s Carer’s Quality of Life Inventory
BASC: Brief Assessment Scale for Caregivers
CFA: Confirmatory factor analysis
CFI: Comparative Fit Index
CGQOL: Caregiver-targeted Quality of Life Measure
CRA: Caregiver Reaction Assessment
DL: Impact on Daily Life
EM: Experience & Meaning
FAST: Functional Assessment Staging Test
FW: Financial Well-being
ICC: Intraclass correlation coefficient
MW: Mental Well-being
NPI: Negative Personal Impact
PPI: Positive Personal Impact
PW: Physical Well-being
PWD: Persons with dementia
QOL: Quality of life
RMSEA: Root Mean Square Error of Approximation
SCQOLS: Singapore Caregiver Quality of Life Scale
WLSMV: Weighted Least Squares method for data with Missing Values
