# Supplementary material for: Development and evaluation of the Singapore Caregiver Quality of Life Scale - Dementia
Source: J Patient Rep Outcomes. 2020 Oct 19;4:84. doi: 10.1186/s41687-020-00252-3 (PMC7572987; doi:10.1186/s41687-020-00252-3)
Supplement: Supplementary file 1 — Additional file 1: Online Supplementary Material S1. Factor loadings in confirmatory factor analysis of a 5-factor model, with each item loaded on one factor. [file 41687_2020_252_MOESM1_ESM.docx]

**Online Supplementary Material S1** Factor loadings in confirmatory factor analysis of a 5-factor model, with each item loaded on one factor ^a^

|  |  | PW | MW | EM | DL | FW |
| --- | --- | --- | --- | --- | --- | --- |
| PW1 | Difficulty falling asleep | 0.72 |  |  |  |  |
| PW2 | Physically tired | 0.76 |  |  |  |  |
| PW3 | Mentally exhausted | 0.77 |  |  |  |  |
| PW4 | Aches and pains | 0.79 |  |  |  |  |
| PW5 | Injury | 0.64 |  |  |  |  |
| PW6 | Poor appetite | 0.85 |  |  |  |  |
| PW7 | Weight loss | 0.84 |  |  |  |  |
| PW8 | Body has weakened | 0.91 |  |  |  |  |
| PW9 | Neglected own medical condition | 0.76 |  |  |  |  |
| PW10 | Sleep well | 0.37 |  |  |  |  |
| PW11 | Difficulty remembering things | 0.85 |  |  |  |  |
| PW12 | Hard to concentrate | 0.72 |  |  |  |  |
| MW1 | Constantly worried |  | 0.62 |  |  |  |
| MW2 | Fearful of losing the patient |  | 0.39 |  |  |  |
| MW3 | Feel sad |  | 0.58 |  |  |  |
| MW4 | No hope |  | 0.86 |  |  |  |
| MW5 | Nobody can help me |  | 0.85 |  |  |  |
| MW6 | Feel guilty |  | 0.56 |  |  |  |
| MW7 | Feel angry |  | 0.85 |  |  |  |
| MW8 | Feel frustrated |  | 0.92 |  |  |  |
| MW9 | Unfair that my family member is sick |  | 0.43 |  |  |  |
| MW10 | No choice but to accept |  | 0.32 |  |  |  |
| MW11 | Taken advantage of |  | 0.45 |  |  |  |
| MW12 | Do something unsafe |  | 0.57 |  |  |  |
| MW13 | Fall and hurt |  | 0.63 |  |  |  |
| MW14 | Embarrassed by the behavior |  | 0.52 |  |  |  |
| MW15 | Worry unable to take care of |  | 0.71 |  |  |  |
| MW16 | Has become a stranger |  | 0.57 |  |  |  |
| MW17 | Societal attitude |  | 0.73 |  |  |  |
| MW18 | Burden to me |  | 0.84 |  |  |  |
| EM1 | Competent as a caregiver |  |  | 0.54 |  |  |
| EM2 | Feel appreciated as a caregiver |  |  | 0.67 |  |  |
| EM3 | Hopeful condition well-managed |  |  | 0.72 |  |  |
| EM4 | Thankful for good things |  |  | 0.82 |  |  |
| EM5 | Make the best of whatever comes |  |  | 0.81 |  |  |
| EM6 | Get satisfaction from caregiving |  |  | 0.83 |  |  |
| EM7 | Experienced positive changes |  |  | 0.79 |  |  |
| EM8 | Support from family |  |  | 0.76 |  |  |
| EM9 | Support from friends |  |  | 0.44 |  |  |
| EM10 | Support from religious group |  |  | 0.49 |  |  |
| EM11 | Family closer together |  |  | 0.75 |  |  |
| EM12 | Caregiver role appreciated by family |  |  | 0.86 |  |  |
| EM13 | Sufficient practical support |  |  | 0.34 |  |  |
| EM14 | Receive useful information |  |  | 0.47 |  |  |
| EM15 | Faith helps me cope |  |  | 0.63 |  |  |
| EM16 | Caretaking bring us closer |  |  | 0.77 |  |  |
| DL1 | Change future plans |  |  |  | 0.86 |  |
| DL2 | Not able to leave home or hospital |  |  |  | 0.82 |  |
| DL3 | Not satisfied with time to myself |  |  |  | 0.96 |  |
| DL4 | No time for recreational activities |  |  |  | 0.92 |  |
| DL5 | Not able to do what I want |  |  |  | 0.95 |  |
| DL6 | Too many things to handle |  |  |  | 0.79 |  |
| DL7 | Work performance affected |  |  |  | 0.88 |  |
| DL8 | Career development affected |  |  |  | 0.94 |  |
| DL9 | Change in work arrangements |  |  |  | 0.90 |  |
| DL10 | Neglected other family members |  |  |  | 0.49 |  |
| DL11 | Disagreements with family |  |  |  | 0.57 |  |
| DL12 | Less time on social activities |  |  |  | 0.83 |  |
| DL13 | Lost contact with friend |  |  |  | 0.76 |  |
| FW1 | Depleting savings |  |  |  |  | 0.96 |
| FW2 | Difficulty to get financial help |  |  |  |  | 0.88 |
| FW3 | Uncertain about future financial situation |  |  |  |  | 0.92 |
| FW4 | Personal spending restricted |  |  |  |  | 0.91 |

^a^ PW: Physical Well-being; MW: Mental Well-being; EM: Experience & Meaning; DL: Impact on Daily Living; FW: Financial Well-being.
