# Supplementary material for: Development and evaluation of the Singapore Caregiver Quality of Life Scale - Dementia
Source: J Patient Rep Outcomes. 2020 Oct 19;4:84. doi: 10.1186/s41687-020-00252-3 (PMC7572987; doi:10.1186/s41687-020-00252-3)
Supplement: Supplementary file 2 — Additional file 2: Online Supplementary Material S2. Spearman’s correlation coefficients between each item and its belonging domain and other domains. [file 41687_2020_252_MOESM2_ESM.docx]

**Online Supplementary Material S2**. Spearman’s correlation coefficients between each item and its belonging domain and other domains. ^a,b^

| PW MW EM DL FW

--------+---------------------------------------------

PW1 | 0.4174 0.1883 0.1687 0.0846 0.0900

PW2 | 0.7625 0.4802 0.1569 0.4607 0.3253

PW3 | 0.7299 0.5064 0.1328 0.3882 0.2944

PW4 | 0.7516 0.4416 0.1420 0.4991 0.3839

PW5 | 0.5112 0.2501 0.0888 0.3176 0.3335

PW6 | 0.7368 0.4647 0.1258 0.4590 0.4841

PW7 | 0.6613 0.3937 0.0372 0.3679 0.3639

PW8 | 0.7969 0.4969 0.0311 0.4910 0.4971

PW9 | 0.6305 0.3679 0.0523 0.4043 0.2550

PW10 | 0.6389 0.4774 0.2027 0.4332 0.3493

PW11 | 0.6397 0.4655 0.2005 0.4185 0.3950

PW12 | 0.6552 0.2990 0.3316 0.3884 0.2636

| PW MW EM DL FW

--------+---------------------------------------------

MW1 | 0.3547 0.6777 -0.0487 0.3595 0.2408

MW2 | 0.2008 0.5055 -0.0616 0.1370 0.1100

MW3 | 0.2580 0.6143 0.0967 0.3702 0.2927

MW4 | 0.5455 0.6169 0.1980 0.4075 0.3758

MW5 | 0.5472 0.7150 0.2227 0.5169 0.4816

MW6 | 0.3310 0.6735 0.0864 0.2950 0.2438

MW7 | 0.5035 0.6882 0.1647 0.5706 0.3261

MW8 | 0.6169 0.7525 0.1758 0.5811 0.4050

MW9 | 0.3421 0.4885 0.1875 0.1750 0.2212

MW10 | 0.2696 0.3105 0.1867 0.1978 0.1200

MW11 | 0.2109 0.5535 -0.0474 0.3208 0.2505

MW12 | 0.2801 0.6578 0.0137 0.3853 0.2566

MW13 | 0.2773 0.5835 -0.0380 0.4552 0.2578

MW14 | 0.2062 0.2862 0.3129 0.2069 0.1960

MW15 | 0.4599 0.6411 0.1040 0.4713 0.4346

MW16 | 0.2969 0.5919 0.2142 0.3713 0.3648

MW17 | 0.4138 0.6160 0.2983 0.4594 0.4334

MW18 | 0.3537 0.5804 0.3581 0.5783 0.5214

| PW MW EM DL FW

--------+---------------------------------------------

EM1 | -0.0358 -0.0077 0.5433 0.0468 0.1365

EM2 | 0.2238 0.1061 0.6616 0.0971 0.1115

EM3 | 0.0773 -0.0209 0.6508 -0.0628 -0.0127

EM4 | 0.1992 0.1719 0.6382 0.1108 0.1076

EM5 | 0.0778 0.0388 0.6922 -0.0301 0.0115

EM6 | 0.1585 0.1652 0.7867 0.1719 0.0164

EM7 | 0.2072 0.0564 0.6874 0.1646 0.0823

EM8 | 0.3156 0.1903 0.6342 0.2096 0.3608

EM9 | -0.0464 -0.0088 0.4862 0.0145 0.1452

EM10 | -0.0219 0.1376 0.5670 0.0674 0.0641

EM11 | 0.2450 0.1036 0.6752 0.2573 0.2261

EM12 | 0.3104 0.2597 0.6850 0.2891 0.3066

EM13 | -0.0653 0.0641 0.3925 -0.0768 0.0880

EM14 | 0.0680 0.1617 0.5295 0.0311 0.1192

EM15 | -0.0223 0.1366 0.6900 0.0524 -0.0415

EM16 | 0.1661 0.0873 0.7007 0.1070 0.0422

| PW MW EM DL FW

--------+---------------------------------------------

DL1 | 0.4023 0.5919 0.1510 0.8027 0.4386

DL2 | 0.4443 0.5005 0.1020 0.7224 0.3892

DL3 | 0.4685 0.4871 0.1640 0.8667 0.4799

DL4 | 0.4364 0.4800 0.1839 0.8433 0.3695

DL5 | 0.4644 0.5241 0.2196 0.9000 0.4367

DL6 | 0.4451 0.4582 0.0769 0.7683 0.3974

DL7 | 0.5681 0.5230 0.2439 0.7633 0.5429

DL8 | 0.5098 0.5553 0.2202 0.8159 0.6448

DL9 | 0.4732 0.5907 0.2289 0.7970 0.5593

DL10 | 0.2393 0.3515 0.0574 0.4632 0.1714

DL11 | 0.4489 0.4530 0.0081 0.4439 0.2700

DL12 | 0.4094 0.4568 0.0075 0.8117 0.4654

DL13 | 0.4000 0.3816 0.1657 0.7000 0.4010

| PW MW EM DL FW

--------+---------------------------------------------

FW1 | 0.5234 0.4776 0.1539 0.5882 0.8708

FW2 | 0.4878 0.4740 0.1324 0.4981 0.8979

FW3 | 0.4071 0.5124 0.2041 0.5082 0.9090

FW4 | 0.4060 0.4390 0.2154 0.5004 0.9266

^a^ PW: Physical Well-being; MW: Mental Well-being; EM: Experience & Meaning; DL: Impact on Daily Living; FW: Financial Well-being.

^b^ Description of individual items can be found in Online Supplementary Materials S1.
